# Rotational direction of flagellar motor from the conformation of FliG middle domain in marine *Vibrio*

**DOI:** 10.1038/s41598-018-35902-6

**Published:** 2018-12-12

**Authors:** Tatsuro Nishikino, Atsushi Hijikata, Yohei Miyanoiri, Yasuhiro Onoue, Seiji Kojima, Tsuyoshi Shirai, Michio Homma

**Affiliations:** 1 Division of Biological Science, Graduate School of Science, Nagoya University, Chikusa-ku, Nagoya 464-8602 Japan; 2 Structural Biology Research Center, Graduate School of Science, Nagoya University, Chikusa-ku, Nagoya 464-8602 Japan; 3 Department of Bioscience, Nagahama Institute of Bio-Science and Technology, 1266 Tamura, Nagahama, 526-0829 Japan; 4 Research Center for State-of-the-Art Functional Protein Analysis, Institute for Protein Research, Osaka University, 3-2 Yamadaoka, Suita, Osaka 565-0871 Japan

## Abstract

FliG, which is composed of three distinctive domains, N-terminal (N), middle (M), and C-terminal (C), is an essential rotor component that generates torque and determines rotational direction. To determine the role of FliG in determining flagellar rotational direction, we prepared rotational biased mutants of *fliG* in *Vibrio alginolyticus*. The E144D mutant, whose residue is belonging to the EHPQR-motif in FliG_M_, exhibited an increased number of switching events. This phenotype generated a response similar to the phenol-repellent response in chemotaxis. To clarify the effect of E144D mutation on the rotational switching, we combined the mutation with other *che* mutations (G214S, G215A and A282T) in FliG. Two of the double mutants suppressed the rotational biased phenotype. To gain structural insight into the mutations, we performed molecular dynamic simulations of the FliG_MC_ domain, based on the crystal structure of *Thermotoga maritima* FliG and nuclear magnetic resonance analysis. Furthermore, we examined the swimming behavior of the *fliG* mutants lacking CheY. The results suggested that the conformation of FliG in E144D mutant was similar to that in the wild type. However, that of G214S and G215A caused a steric hindrance in FliG. The conformational change in FliG_M_ triggered by binding CheY may lead to a rapid change of direction and may occur in both directional states.

## Introduction

Bacteria move in various environments by rotating flagellar filaments in either counterclockwise (CCW) or clockwise (CW) direction. The flagellar motor, which is present at the base of the filament and is embedded in the membrane, can rotate by conversion of the electrochemical potential across the membrane. It is known that *Escherichia coli* and *Salmonella enterica* have H^+^-driven lateral (or peritrichous) flagella, and *Vibrio alginolyticus* has an Na^+^-driven polar flagellum as well as H^+^-driven lateral flagella^1^. The motor is composed of two parts, the stator and rotor, and torque is generated by interaction between the stator and rotor. The stator functions as an ion channel to convert the ion flux into mechanical forces.

In *V. alginolyticus*, the Na^+^-driven stator is composed of two membrane proteins, PomA and PomB, which assemble in a 4 PomA: 2 PomB stoichiometry^2^. It is known that PomA and PomB are orthologs of MotA and MotB, respectively, in the H^+^-driven motor of *E. coli* and *S. enterica*^3–5^. The stators can be activated by interacting with the C ring composed of cytoplasmic rotor components^6–8^. The C ring consists of three proteins (FliG, FliM, and FliN) and is important for torque generation and determination of the rotational direction^9^. FliM and/or FliN interact with CheY, one of the chemotaxis signaling factors, and switch the flagellar rotational direction. The phosphorylated CheY (CheY-P) can bind FliM and/or FliN to switch the rotational direction of flagellar motor from the default CCW to CW rotation. CheY-P is automatically dephosphorylated by CheZ, which is the other component of chemotactic signal factor, and CheY dissociates from FliM and/or FliN^10,11^.

The full-length structure of FliG was described in *Aquifex aeolicus*^12^. There are three distinctive domains in FliG: N-terminal domain (FliG_N_), middle domain (FliG_M_), and C-terminal domain (FliG_C_). Each domain has an Armadillo repeat motif (ARM): ARM_N_, ARM_M_ and ARM_C_, respectively^12–14^. The domains have tandem repeats of several hydrophobic residues in α-helices. Several ARMs were also found in the crystal structure of β-catenin, suggesting that it is important for intermolecular interaction^15^. The hydrophobic residues of ARM in FliG are more conserved than those of β-catenin; moreover, the crystal structure of β-catenin, ARM_M_ and ARM_C_ were well aligned^14^. FliG_N_ works as the anchor of the C ring by interacting with the MS ring, which is composed of FliF and is buried in the inner membrane^16^. It has been shown that ARM_N_ is important for intermolecular interaction with FliG_N_ to properly assemble the MS-C ring^17,18^. FliG_M_ interacts with FliM, which receives the chemotaxis signals transmitted by CheY binding^19,20^. Conserved charged residues in FliG_C_ electrostatically interact with those in cytoplasmic loops of PomA (MotA), thereby generating torque^21–23^. It has been suggested that the conformation of the linker region between FliG_M_ and FliG_C_, called Helix_MC_, is important for determining the rotational direction or switching. A three-amino-acid deletion mutant (ΔPAA mutant) in Helix_MC_ of *Salmonella* drove CW-locked rotation. It was deduced that the mutation stabilized the intramolecular interaction between FliG_M_ and FliG_C_, and induced a conformational change in the Helix_MC_ region to affect the CheY binding to FliM^13,24,25^. In contrast, the three-amino-acid deletion mutant (ΔPSA) of *V. alginolyticus* FliG corresponding to the *Salmonella* ΔPAA mutant conferred no flagellation phenotype, presumably because of defects in flagellin transport^26^. The C ring is also a transport machinery for flagellin. The change in the Helix_MC_ region affects the interaction between the two ARMs of FliG: ARM_M_ and ARM_C_. The interaction between ARM_M_ and ARM_C_ is similar to the intermolecular interaction with ARM_N_^17,18^. To change the C ring state, ARM_M_ interacts with intramolecular or intermolecular ARM_C_, and FliM affects the FliG structure of Helix_MC_^12,19,27^. When Helix_MC_ is disordered, FliG folds into the compact structure by the interaction between ARM_M_ and ARM_C_ in CW rotation. In contrast, because of the stable α helix, ARM_M_ of the neighbor molecule interacts with ARM_C_ so that the distance between FliG_M_ and FliG_C_ is extended in CCW rotation. However, the correlation between the structure of FliG and rotational direction is still unclear.

**Figure 1:**
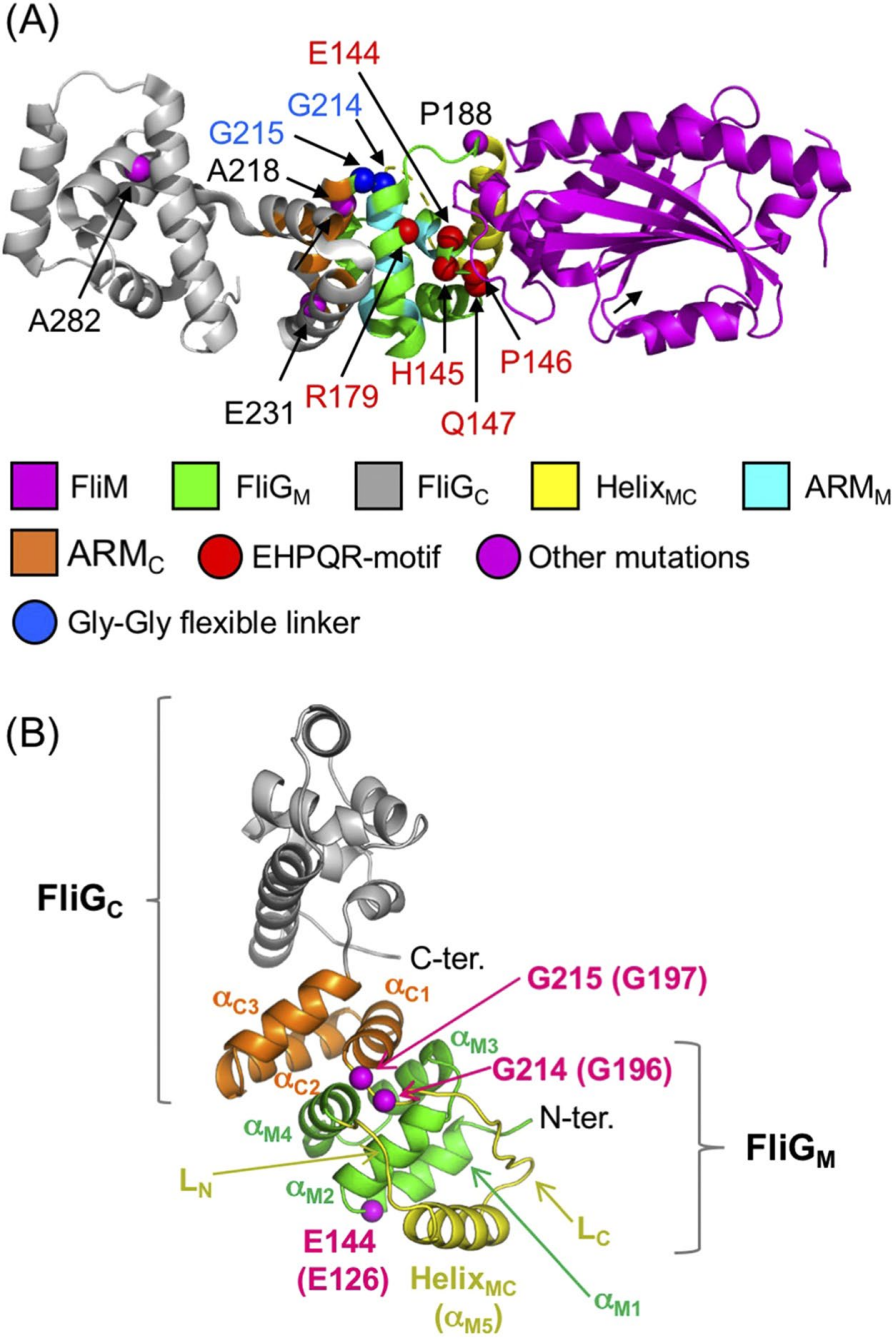
A structural model of the mutations in this study. (**A**) The structure of the FliG-FliM complex from *Thermotoga maritima* (PDB code 4FHR). FliG shows two distinct domains, FliG_M_ and FliG_C_. The ARM_M_ and ARM_C_ domains are a part of FliG_M_ and FliG_C_, respectively. The Helix_MC_ and the Gly-Gly flexible linker region connect between FliG_M_ and FliG_C_. The structures of FliG_M_, FliG_C_, Helix_MC_, ARM_M_, ARM_C_ and FliM are shown in green, gray, white, cyan, orange and magenta, respectively, as a ribbon model. The atoms of blue, red, and magenta balls show Gly-Gly flexible linker (G214 and G215 residues), residues at the EHPQR motif (E144, H145, P146, Q147, and R179) and the residues of other point mutations, respectively, in *Vibrio alginolyticus*. The hydrophobic residues of the ARM_M_ and ARM_C_ were determined by the alignment of the structure and amino acid sequence between β-catenin, FliG_M_ and FliG_C_, respectively^15^. (**B**) The architecture of the FliG protein is divided into two domains: FliG_M_ and FliG_C_. The FliG_MC1_ domain (D113 through M235 of *T. maritima*) was used for the molecular dynamics simulation. FliG_M_, FliG_C_ and Helix_MC_ are colored green, orange, and yellow, respectively. In this study, the C-terminal region and N-terminal region of Helix_MC_ are called L_C_ and L_N_, respectively. E144, G214, and G215 mutation residues of *V. alginolyticus* (E126, G196, and G197 of *T. maritima*, respectively) are shown as spheres in magenta.

We have isolated and characterized five rotational mutants of the *fliG* gene of *V. alginolyticus*: two CCW-biased, two CW-biased, and one CW-locked^28^. Among the mutants, the G214S and G215A mutants showed CCW-biased and CW-locked rotations, respectively, and these mutations were located in the hinge linker between FliG_M_ and FliG_C_, called Gly-Gly flexible linker (Fig. 1). It was speculated that the bending at Gly-Gly flexible linker depends on the conformation of the Helix_MC_. The G214 and G215 residues, which were located next to each other, produced the opposite phenotypes for the rotational bias. This indicated that the orientation of the FliG_C_ domain was determined by the state of the Gly-Gly flexible linker. The A282T mutation in FliG_C_ conferred the CW-biased phenotype. The structure of FliG_C_ should be fixed to the CW conformation stabilized by the interaction between helix α1 and helix α3 of FliG_C_ as suggested by NMR experiments and molecular dynamics (MD) simulation^29^.

In this study, we isolated the E144D mutant that increased the switching frequency of the rotational direction (Figs 1 and S1). E144 residue is highly conserved among the species and belonging to the EHPQR-motif which is the FliM binding site in FliG_M_. To characterize this mutant, we combined 3 mutations (the G214S, G215A, and A282T mutations that showed CCW-biased, CW-locked, and CW-biased rotations, respectively) with the E144D mutation. To elucidate how the conformational change occurred in the 3 mutants (E144D, G214S, and G215A), we performed MD simulation using a co-crystal structure of FliG and FliM in *Thermotoga maritima* and NMR data was obtained from 2D ^1^H-^15^N TROSY HSQC spectra of the *Vibrio* FliG_MC_ fragment. We observed the rotational direction and the rotational switching of the *Vibrio fliG* mutants in the absence of CheY to investigate the effect of CheY on *fliG* mutants. Here, we discuss the association of the conformational change of FliG_C_ and interaction between FliM and FliG_M_.

## Results

### Analysis of point mutants of *Vibrio fliG*

**Figure 2:**
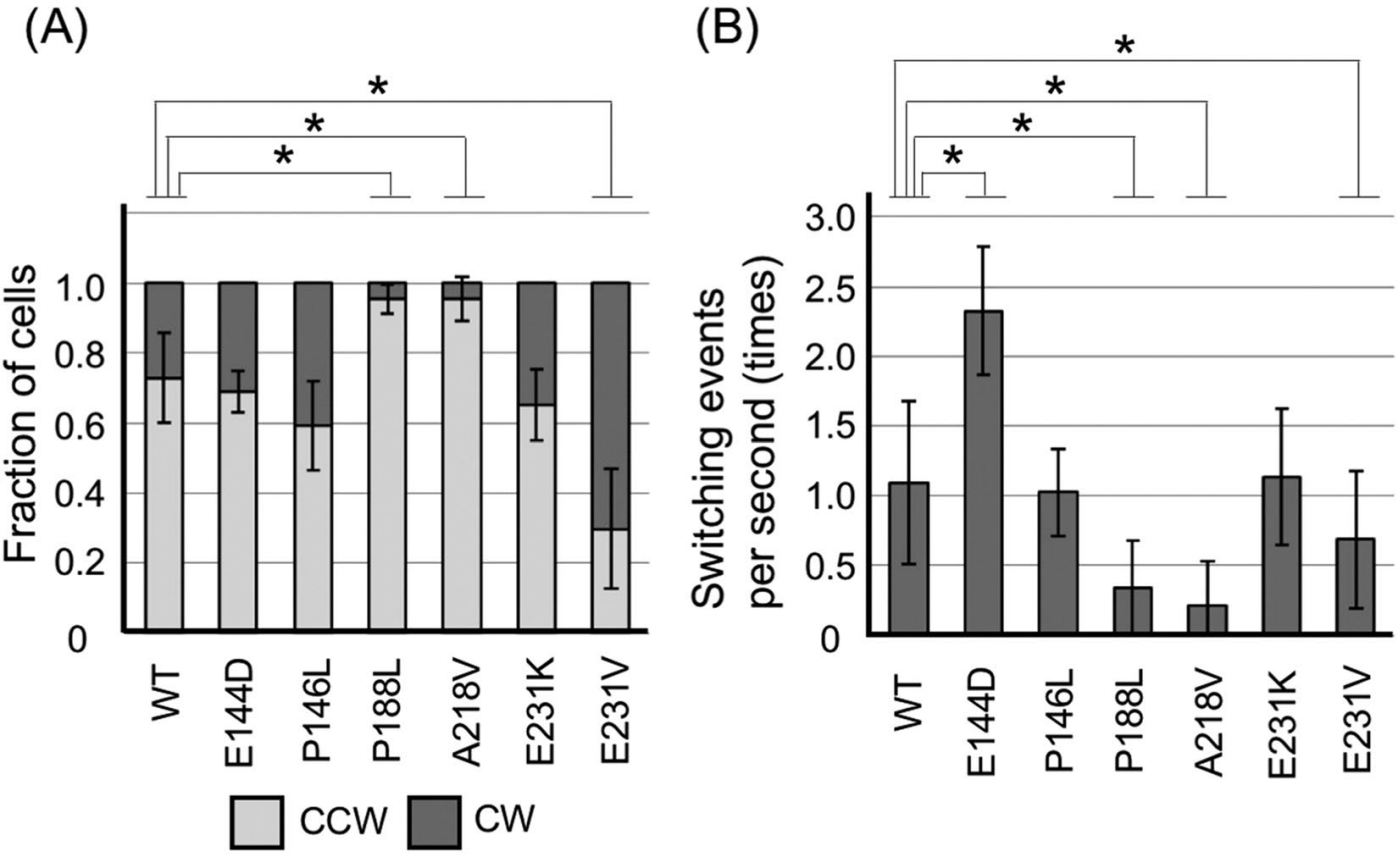
The biased directional rotation of *fliG* mutants. The *fliG/pomA/pomB* mutant (NMB301) harboring pNT1 with a *fliG* mutation and pYA303 with a wild-type (WT)-*pomA* and *pomB* was grown and observed by high-intensity dark-field microscopy. (**A**) The ratio of counterclockwise (CCW) rotation to CW rotation of the *fliG* mutants is shown in the column. All the experiments were repeated at least 6 times, and average values with standard deviation (SD) are shown. (**B**) The switching frequency in 10 sec was measured. All the experiments were repeated at least 6 times, and average values with SD are shown. The black bars indicate that *P* < 0.01 (*) for WT-FliG versus the mutants by the Welch’s t-test.

In previous studies, we generated various point mutants of *fliG* in *Vibrio*^8,28^ based on the phenotype of *E. coli* and *S. enterica* mutants^30–32^. We generated additional six mutants of *Vibrio* FliG: E144D, P146L, P188L, A218V, E231V, and E231K; where these residues are highly conserved among the species. The E144D, P188L, A218V and E231K mutants showed CCW-biased rotation, whereas P146L and E231V mutants showed CW-biased rotation in the corresponding mutants of *E. coli* and *S. enterica*. We investigated the rotational direction and switching events (Fig. 2A). The wild-type (WT)-FliG had a CW: CCW ratio of 25: 75 and switching events of 10 times per 10 sec as previously reported^28^. P146L and E231K mutants showed phenotypes similar to that of WT. The E231V mutant showed CW-biased rotation (CW: CCW ratio of 70: 30, *P* > 0.01) and seven instances of direction-switching per 10 sec; however, the CW-biased rotation was not as significant as that in the Q147H and G215A mutants. P188L and A218V mutants showed strong CCW-biased rotation (CW: CCW ratio of 5: 95 (*P* > 0.01) and 5: 95 (*P* > 0.01), respectively) and decreased switching events (three times and two times per 10 sec, respectively (*P* > 0.01)). The E144D mutant showed the same rotational bias as that of WT, but the switching frequency was twice that of the switching frequency in the WT (i.e., 20 times per 10 sec, *P* > 0.01). Such frequent rotational switching was observed when the repellent (phenol) was added to WT *Vibrio*^33^.

### Analysis of the mutants in the *fliG* EHPQR motif

To further investigate the role of the E144 residue in determining the rotational direction, we generated seven mutations (E144A, E144N, E144S, E144Q, E144W, H145E, and H145W) in the EHPQR motif. The expressions of the FliG protein in all mutants were detected (Fig. S2). We performed the motility assay on soft-agar plate, to evaluate the bacterial motility (Fig. S3A,B). WT-FliG formed a large motility ring, and no rings were observed in the vector control. The small motility ring was observed in the E144D and E144N mutants, but not in the E144A, E144Q, E144S, E144W, H145E, and H145W mutants.

To reveal whether declining or disappearance of motility were because of defects in flagellum formation, we counted polar flagella by immunostaining (Fig. S3C). The ratio of flagellation of WT-FliG was 28% in the total cells but that of the vector control was zero. The ratios of E144D, E144Q, and E144N mutants, which formed a small motile ring, were 14%, 9%, and 6%, respectively. The ratios of E144A, E144S, E144W, and H145E mutants, which formed no rings, were 7%, 6%, 8%, and 5%, respectively. We could not detect flagella in the H145W mutants. These results indicated that those mutations of the EHPQR motif only partially inhibited flagellar formation.

We observed the motile fraction by dark-field microscopy and measured the ratio of motile cells and non-motile cells (Fig. S3D). The motile fraction in WT was 41%. E144D, E144N, and E144Q mutants had motile fractions of 38%, 4% and 1%, respectively. E144A, E144S, E144W, H145E, and H145W mutants were not motile. The mutations in the EHPQR motif affected motility as well as flagellar formation.

### Effects of the E144D mutation on the rotational direction and switching events

**Figure 3:**
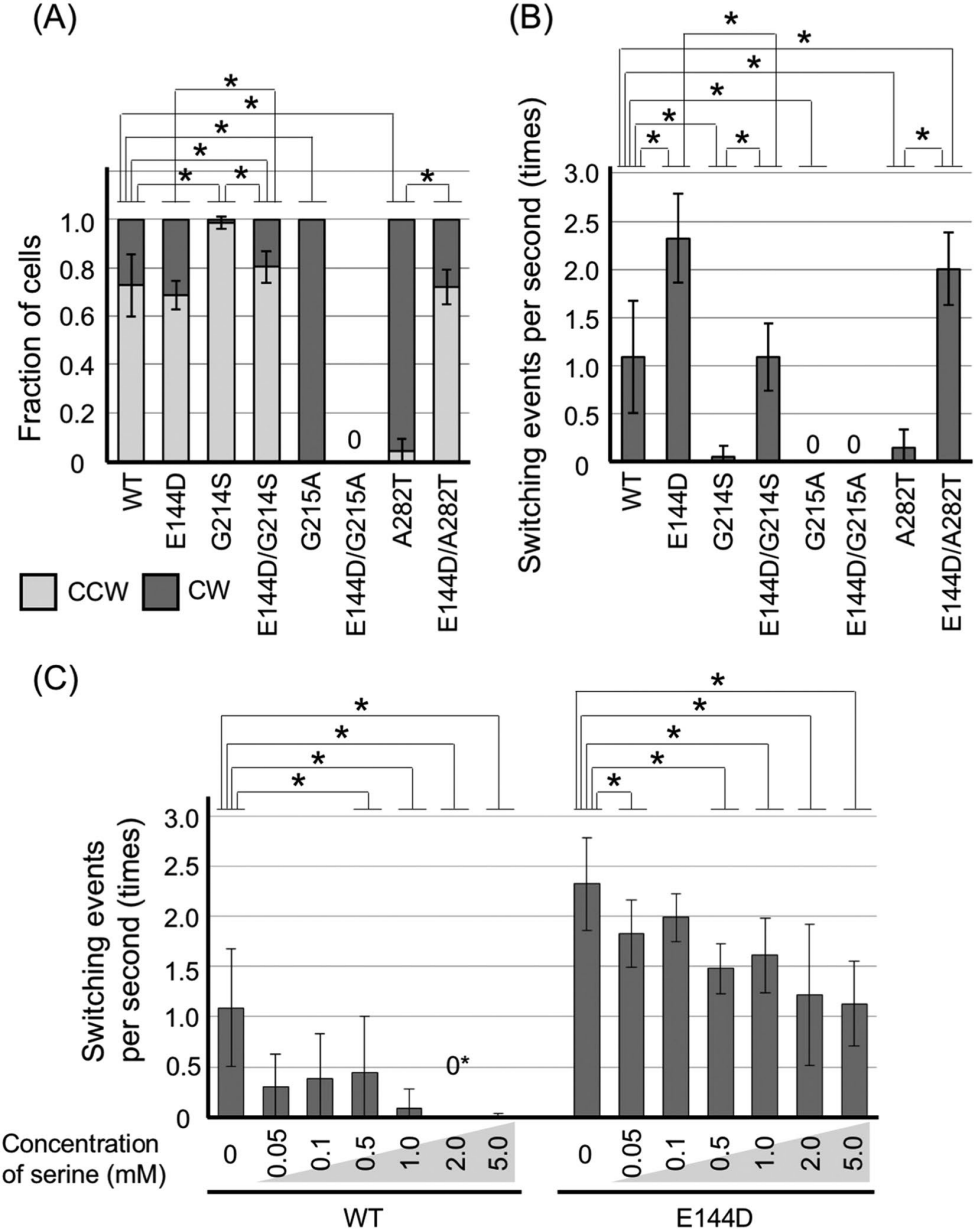
Characterizations of the E144D mutant in *Vibrio alginolyticus*. The *fliG/pomA/pomB* mutant (NMB301) harboring pNT1 with a *fliG* mutation and pYA303 with a wild-type (WT)-*pomA* and *pomB* and the *fliG* mutant (NMB198) harboring pNT1 with a *fliG-WT* or *fliG-E144D* mutation were grown and observed by high-intensity dark-field microscopy. (**A**) The ratio of counterclockwise (CCW) rotation and clockwise (CW) rotation of the *fliG* mutations is shown in the column. All the experiments were repeated at least 6 times, and average values with standard deviation (SD) are shown. (**B** and **C**) The switching frequency in 10 sec was measured. All the experiments were repeated at least 6 times, and average values with SD are shown. Data of (**A**) and (**B**) for the WT and E144D mutant are from Fig. 2A,B, respectively. The E144D/G215A mutant did not flagellate, so that it showed 0 on (**A**,**B**). (**C**) The effect of serine in the WT-FliG and E144D mutant. To ignore the effect of chemotactic adaptation, the switching events were observed within 3 min after addition of serine. Serine was added to a final concentration of 0.05, 0.1, 0.5, 1.0, 2.0 and 5.0 mM. 0* in the WT with 2.0 mM serine indicates that few cells switched their rotational direction. The WT and E144D mutant without serine are from the WT and E144D mutant of Fig. 2B, respectively. All the experiments were repeated at least 6 times, and average values with SD are shown. The black bars indicate that P < 0.01 for WT-FliG versus the mutants (**A**,**B**) or the V buffer background versus the addition of serine with the V buffer (**C**) by the Welch’s t-test.

To elucidate the effect of the E144D mutation on the conformation of FliG, we generated double mutants of *fliG*. In our previous report, some *fliG* mutants showed CW-biased or CCW-biased phenotypes^28^. We selected three mutations, G214S, G215A, and A282T (Figs 1A and S1), and they showed CCW-biased, CW-locked, and CW-biased rotations respectively. Since the G214S and G215A mutations were located in the Gly-Gly flexible linker, we speculated that the mutations fixed the conformation of FliG_C_ either in CCW state or CW state. In contrast, the A282T mutant tended to change the CW conformation in FliG_C_, as suggested by MD simulations and NMR experiments^29^. We combined these mutations with the E144D mutation (E144D/G214S, E144D/G215A, and E144D/A282T mutants) and measured the expression level of FliG. Similar amounts of FliG were detected in all the mutants (Fig. S2). We observed the rotational direction and switching events using high-intensity dark-field microscopy (Fig. 3A,B). E144D/G214S and E144D/A282T mutants showed a rotational direction similar to that of the WT (CW: CCW ratios of 20:80 and 30:70, respectively). For the switching events, the E144D/A282T mutant behaved similarly to the E144D single mutant (20 times in 10 sec, with *P* > 0.01 versus WT); however, the E144D/G214S mutant behaved similarly to WT (11 times in 10 sec, *P *> 0.01 versus the E144D mutant). These results indicated that the E144D mutation suppressed the G214S mutation, which conferred the CW state of FliG_C_. On the other hand, the E144D mutation dominated A282T. The E144D/G215A mutant lost flagellar formation (Fla^–^ phenotype), suggesting that the assembly or transport of flagellar proteins was affected by the double mutation.

To clarify how the E144D mutations affected the state of FliM, we investigated the switching events in 10 sec with serine, an attractant of *Vibrio* that induced the CCW rotation. We observed the WT and the E144D mutant in the presence of 0.05, 0.1, 0.5, 1.0, 2.0, and 5.0 mM of serine (Fig. 3C). The WT decreased the switching events as the concentration of serine increased, and the switching was not observed above 1 mM serine. On the other hand, the switching of the E144D mutant also significantly decreased with at least 0.5 mM of serine (*P* > 0.01), but the switching events with 5.0 mM of serine in the E144D mutant were still half of those observed without serine. The E144D mutant seems to be almost insensitive to the chemotactic signal and the motor can still switch the rotational direction under the attractant signal more often compared to the WT.

### MD simulation of FliG mutants showed different fluctuations in Helix_MC_

**Figure 4:**
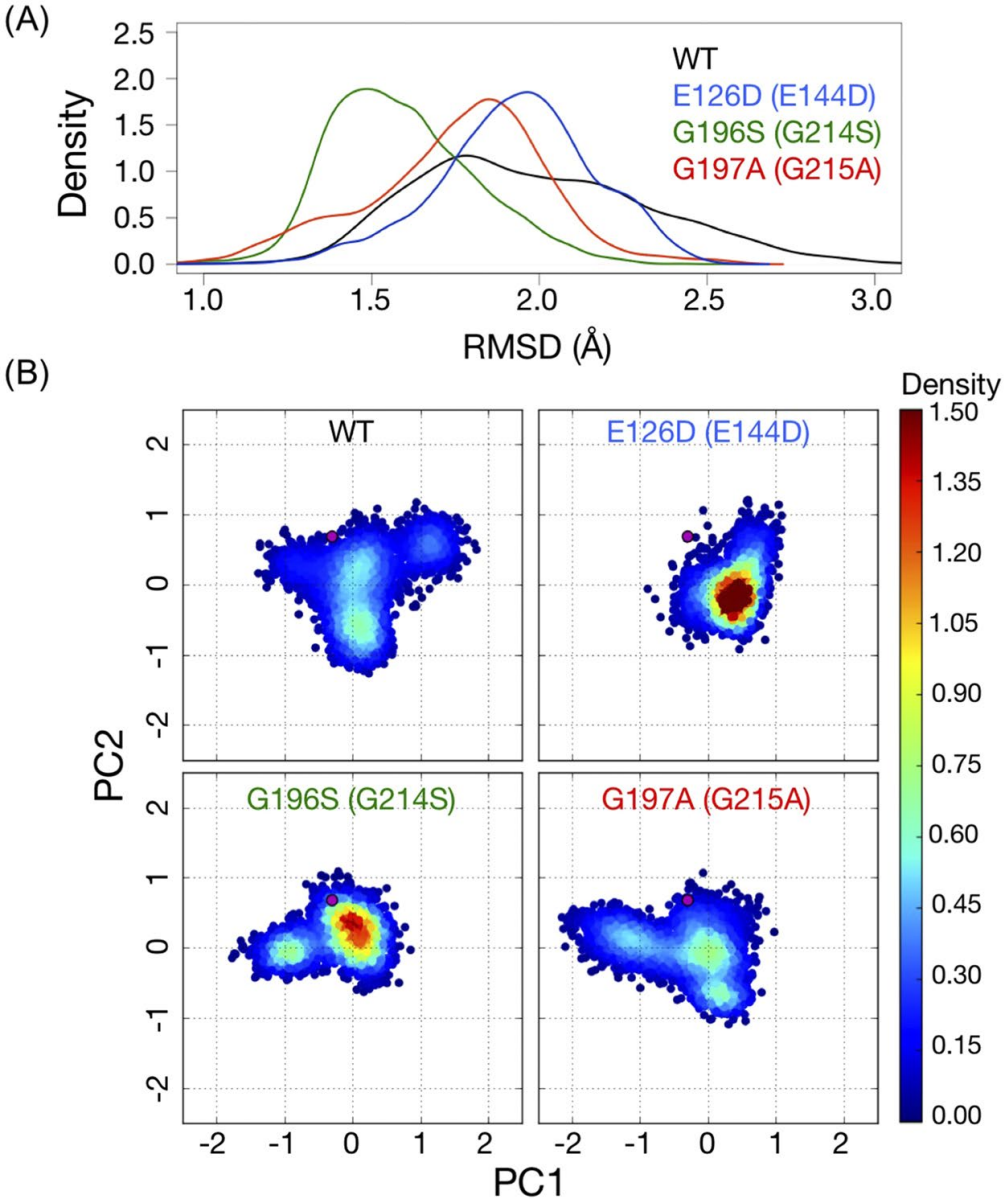
(**A**) The root mean square deviation (RMSD) distributions of the wild-type (WT) and mutant FliG_MC1_ conformations during the simulations compared with the crystal structure of the reference. The horizontal axis shows the RMSD, and the vertical axis shows density of RMSD values. (**B**) Projections of the conformations of each FliG_MC1_ protein in the molecular dynamics trajectories upon the PC1 (horizontal) and PC2 (vertical) axes. For each protein, 3,000 conformations were plotted. The densities of conformation on the plane were estimated by the Gaussian kernel density estimation.

To gain insight into the molecular mechanisms of how the FliG_M_ mutations affected the rotational directions and switching events at atomic resolution, we performed MD simulations of the structures of FliG_MC1_ from *T. maritima* and the three mutants, E126D, G196S and G197A (corresponding to E144D, G214S, and G215A in *Vibrio*). The mean value of the root mean square deviation (RMSD) of the WT FliG_MC1_ from the reference during the simulations was 1.9 Å (SD ± 0.4 Å) and showed a slightly broader distribution compared to those of the three mutants (1.9 Å ± 0.2 Å for E126D, 1.6 Å ± 0.2 Å for G196S, and 1.8 Å ± 0.3 Å for G197A) (Fig. 4A). This indicated that all the proteins maintained their native-like structure during the 100 ns simulations.

Figure 4B shows the projections of the structures of the WT and three mutants during the simulations on to the first (PC1) and second (PC2) components of the principal component analysis (PCA). Compared to the WT, variances for the conformations of E126D mutants along both PC1 and PC2 axes were small, indicating that structural fluctuations in E126D were smaller than those of the WT, even though its major conformation was dispersed from the crystal structure. A similar trend was observed in the G196S mutant, in that, the variances of PC2 of the mutant were relatively smaller than that of the WT. In the G197A mutant, the variance along the PC2 was similar, but the distribution of conformations along the PC1 slightly differed from that of the WT. These results indicated that single amino-acid substitutions appeared to restrict or change the conformational space observed in the WT.

**Figure 5:**
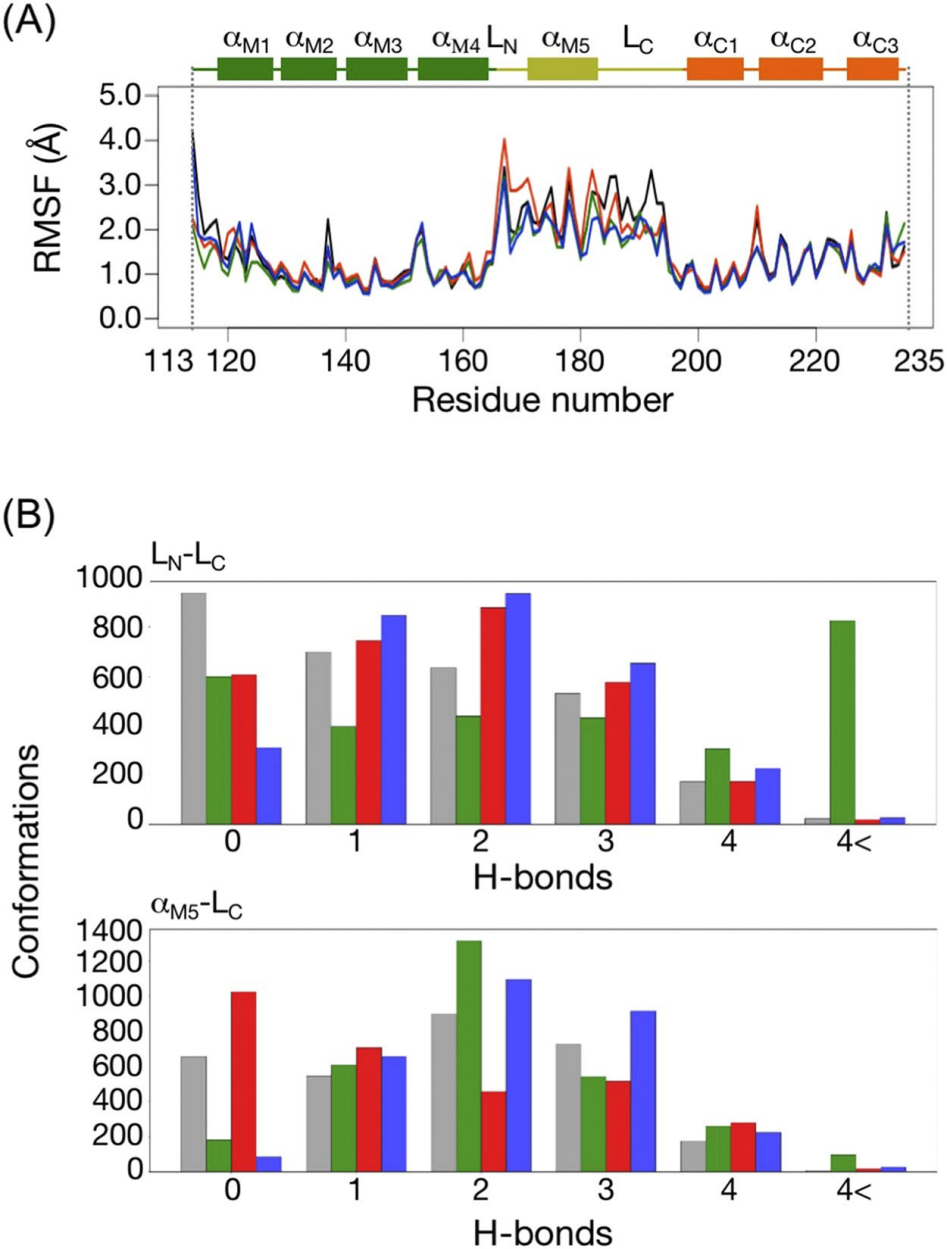
(**A**) The root mean square fluctuation (RMSF) of the residues in the FliG_MC_ domains of the wild-type (WT) and mutant proteins. The secondary structure of the FliG_MC1_ domain colored by the domain architecture is shown on the top of the plot. (**B**) The number of hydrogen bonds formed between L_N_ and L_C_ (upper) and between α_Μ5_-L_C_ (lower) in each conformation of the WT and mutant proteins. The horizontal axis indicates the number of H-bonds, and vertical axis indicates the number of conformations. For each protein, 3000 conformations were used for the calculation. Colors in this figure indicate WT (black), G196S (G214S) (green), G197A (G215A) (red) and E126D (E144D) (blue), respectively.

**Figure 6:**
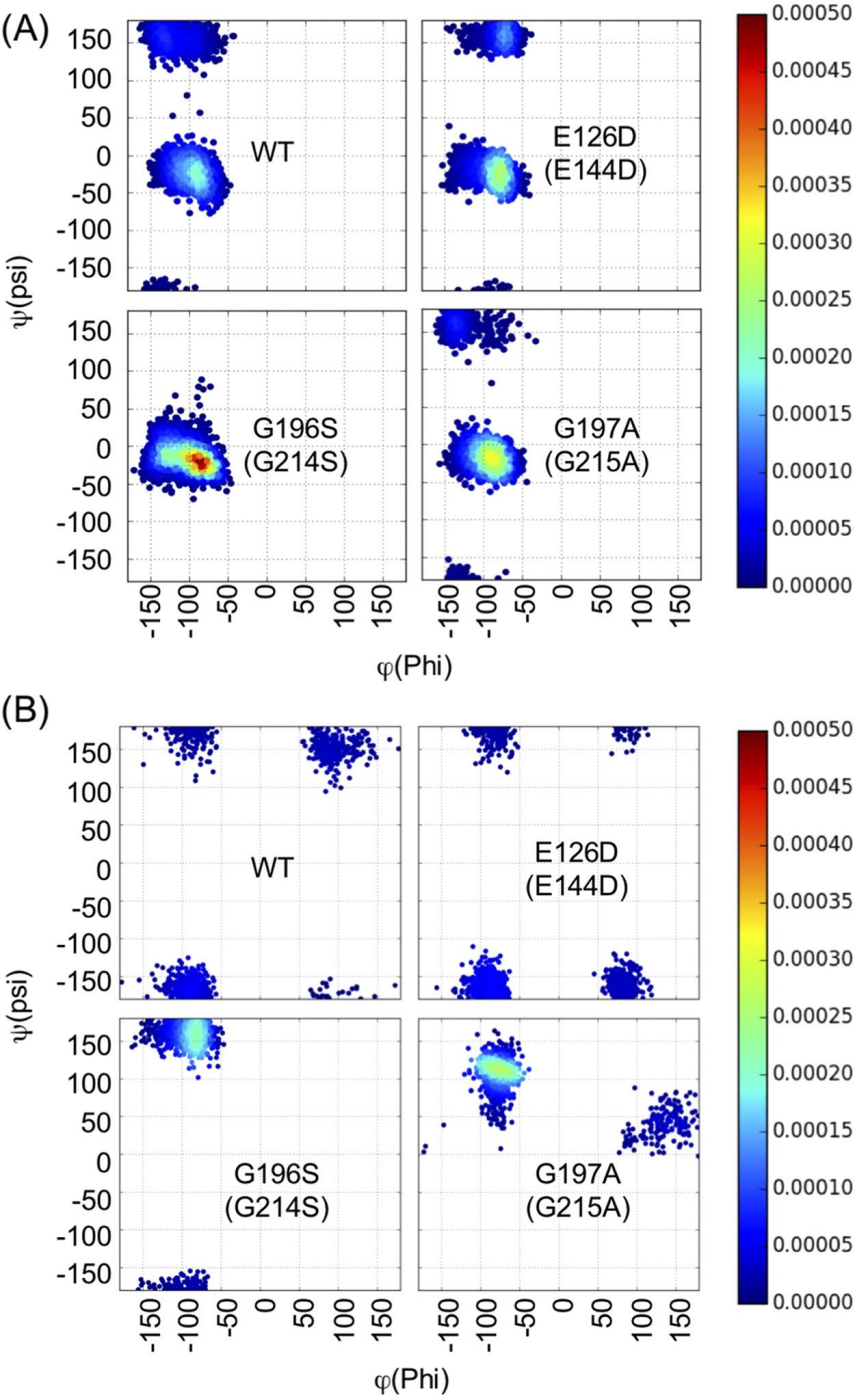
Distribution of the main-chain dihedral angles at position 195 (**A**) and 196 (**B**) on *Thermotoga maritima* FliG_M_. The horizontal axis and vertical axis show the rotatable angles of C_α_-C bond (phi, ϕ) and C_α_-N bond (psi, ψ), respectively. For each protein, 3,000 conformations were calculated. The densities were calculated by Gaussian kernel density estimation.

To reveal the amino-acid residues of FliG_MC1_ that contributed to the difference in the collective motions observed in the PCA, we measured the root mean square fluctuation (RMSF) of each amino-acid residue from the reference structure (Fig. 5A). The regions, which were highly fluctuated were common among the proteins; the Helix_MC_ consisting helix a5 (α_M5_) and two loops (L_N_ and L_C_) in between as a linker helix connecting ARM_M_ and ARM_C_ domains^20^. The degree of fluctuation of the Helix_MC_ differed among the mutants; that of G196S and E126D was smaller, while that of G197A was larger than that in the WT in the L_N_ loop (Fig. 5A). The mutation positions of G196 and G197 corresponding to the Gly-Gly flexible linker were located in the L_C_ of Helix_MC_. The mutations of G196S and G197A conferred a steric hindrance by increasing side chain volumes and affecting the flexibility of the region. The distributions of main chain dihedral angles of psi at position 195 in G196S mutant and at position 196 in G197A mutant, respectively, were confirmed to be altered compared to that in the WT and E126D (Fig. 6), and these alterations contributed to the different perturbations on Helix_MC_.

We found that the conformational alterations of the Helix_MC_ among the proteins were characterized by the number of hydrogen bonds within the Helix_MC_, especially in two parts: 1) between L_N_ and L_C_; 2) α_M5_ and L_C_ (Fig. 5B). Compared to WT, G196S had many hydrogen bonds in the conformations of L_N_-L_C_ and α_M5_-L_C_, suggesting that an increase in the number of hydrogen bonds within the Helix_MC_ contributed to decreased fluctuation of this region in the mutant protein. On the other hand, G197A had a larger number of hydrogen bonds in L_N_-L_C_, but less in α_M5_- L_C_ than that in the WT, reflecting the increase the structural fluctuations in the L_N_ region in the G197A mutant. Interestingly, E126D showed a similar trend in the number of hydrogen bonds to the G196S mutant, although the mutation position was spatially separated from both L_N_ and L_C_. The substitution of Glu to Asp, which has a shorter side chain, might decrease the number of contacts with residues on α_M5_, and simultaneously increase the number of hydrogen bonds between α_M5_ and L_C_. These results suggest that the difference in the conformational dynamics of Helix_MC_ induced by mutations might lead to the difference in rotational direction and switching events.

### Analysis of 2D ^1^H-^15^N TROSY HSQC spectra of *Vibrio* FliG_MC2_ fragment by NMR

**Figure 7:**
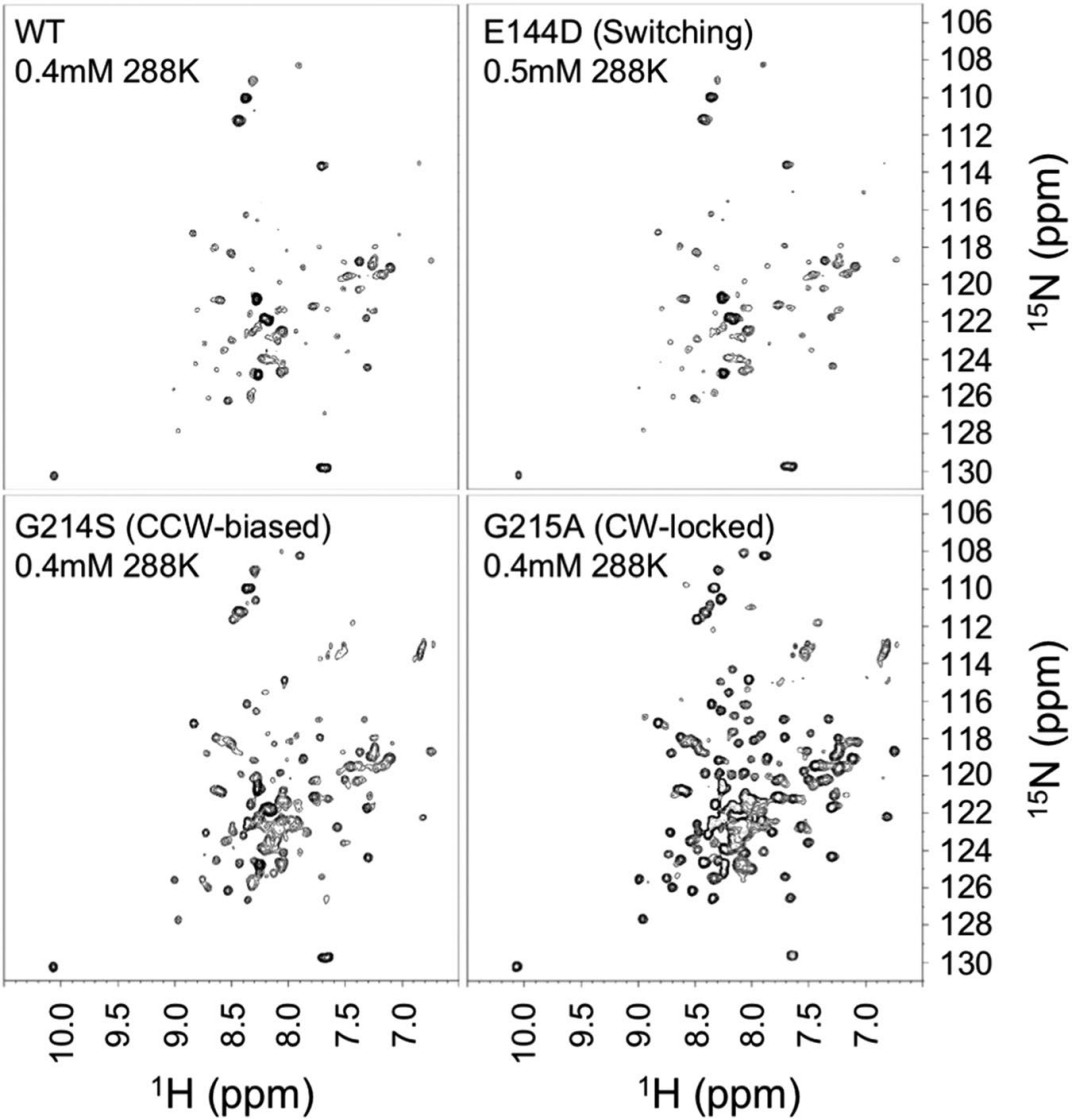
2D ^1^H-^15^N TROSY HSQC spectra of *Vibrio* FliG_MC2_ fragment of the wild-type (WT)-FliG and the E144D, G214S, and G215A mutants. The concentration and measurement temperature of ^15^N labeled FliG_MC_ are shown in each spectrum. The mutant phenotype is shown in the parenthesis of each spectrum. All spectra were measured by Avance-III HD 500 spectrometer equipped with a BBO cryogenic probe.

To support the results of the MD simulation, we obtained 2D ^1^H-^15^N TROSY HSQC spectra of *Vibrio* FliG_MC2_ fragment, the region from G122 through L351, by NMR. We obtained the spectra in five mutations (E144D, Q147H, G214S, G215A, and A218V) (Figs 7 and S4). The spectra of E144D, Q147H, and A218V were similar to that of the WT, suggesting that the conformation of FliG_M_ and FliG_C_ of the E144D mutant did not largely change compared to that of the WT. In contrast, changes in signal intensities and chemical shift were observed for several amide signals in the G214S and G215A mutations, suggesting that the conformation of FliG_M_ and FliG_C_ of G214S and G215A mutants changed. These results suggested that the *in silico* model of the conformational change of FliG by MD simulation in *T. maritima* was supported by the NMR analysis of *Vibrio* FliG_MC2_ fragment.

### The rotational direction and switching events on *fliG* mutants in the *cheY* deletion strain

Since the conformation of FliG depended on CheY binding to FliM, we speculated that the conformational change in *fliG* mutants also affected the state of FliM. We hypothesized that the E144D mutation affected the interaction with FliM transmitting the chemotaxis signal by the CheY binding. Thus, we constructed a *cheY* deletion mutant in the Δ*fliG* strain. We introduced E144D, Q147H, G214S, G215A, and A282T mutations of *fliG* into this strain and observed the rotational direction and switching events by high-intensity dark-filed microscopy (Fig. S5). Q147H mutants show a CW-biased rotation^28^. WT-FliG showed a CCW-locked phenotype in the Δ*cheY* strain. The E144D, Q147H, G214S, and A282T mutants showed CCW-locked rotation in the absence of CheY. These results suggested that the large fluctuation in either CW or CCW state of FliG by the E144D mutation was determined by the interaction between FliG and FliM depending on CheY binding. Furthermore, the CW rotation of these mutants needed the CheY binding. In contrast, G215A mutant showed CW-locked rotation with or without CheY, suggesting that CheY binding to FliM did not affect the flagellar rotation in G215A mutant.

## Discussion

In this study, we isolated 6 *fliG* mutants. Two of them (P188L and A218V) showed CCW-biased flagellar rotation. Surprisingly, although the E144D mutant was similar to the WT in the ratio of the rotational direction, it increased the frequency of switching events from CCW rotation to CW rotation or vice versa. Both the P188L and E144D mutations were expected to confer the CCW-biased rotation of flagella according to a previous mutational analysis of *S. enterica*, in which the E125D and P169L mutations (corresponding to the residues of E144 and P188 of *V. alginolyticus*) were used^30^. We have previously shown that the G204S/A218V mutation conferred the CCW-biased phenotype, and the G204S mutation was similar to the rotational phenotype of WT^28^. We confirmed that the A218V mutation by itself conferred the CCW-biased rotation. The Helix_MC_ contains 88 residues, and the P188L mutation stabilizes α_M5_ in Helix_MC_ as α helix. This assumption was supported by the evidence that the ΔPAA mutation of *S. enterica* conferred the CW-locked phenotype, and this conserved proline residue of P169 in *S. enterica* corresponded to the P188 residue of *V. alginolyticus*^13^. The A218 residue was located on the same side of the Armadillo repeat motifs and the A218V mutation affected the hydrophobic interaction between ARM_M_ and ARM_C_ to stabilize the CCW state structure. The E144D mutation on the EHPQR motif of FliG_M_ was located in the binding site of FliM, affecting both the stability of the α helix in FliG_M_ and the interaction with FliM.

To clarify the effect of the interaction between FliG and FliM on the determination of the flagellar rotation direction, we introduced various mutations in residues on the EHPQR motif of *fliG* in *Vibrio*. The Q147H and R179H mutants showed the CW-biased rotation and Fla^−^ phenotype, respectively^8,28^. We introduced six other mutations on E144 and H145 residues: four of them lacked motility (Mot^−^), and 2 slightly inhibited motility. Moreover, their flagellation rates were slightly reduced, and the H145W mutant was not flagellated at all. These results suggested that mutational effects on the conformational change between CCW and CW states of FliG and/or the interaction with FliM slightly inhibited the flagellar assembly and torque generation. We speculated that these mutations affected the interaction with FliM rather than the conformational change of FliG. In the studies of *E. coli, S. enterica*, and *Helicobacter pylori*, both the rotational biased and Mot^−^ mutants have been isolated, and the mutations were located on the EHPQR motif ^31,34–36^. In some Mot^−^ mutants, the interaction between FliG and FliM was not detected by the pull-down experiments^34^. It was reported that many *fliG* mutants exhibited the Mot^−^ phenotype at high temperatures^32,37^. This may suggest that the structure of FliG is highly mobile and cannot be easily affected by temperature.

In our laboratory collection of *Vibrio* FliG mutants, the G214S, G215A, and A282T conferred CCW-biased, CW-locked, and CW-biased phenotype, respectively, and these mutations have been suggested to affect the stability of the conformation of FliG_C_ to fix either the CCW or CW state^26,27^. To investigate the effect of the E144D mutation, we generated three double mutants (E144D/G214S, E144D/G215A, and E144D/A282T). The E144D/G214S mutation conferred a phenotype similar to that of the WT, the E144D/G215A mutant could not form the polar flagellum, and the E144D/A282T mutation conferred the same phenotype of E144D. These results indicated that the effects of the E144D mutation varied among the G214S, G215A, and A282T mutants. The phenotype of E144D/A282T mutant indicated that the conformational change of FliG_M_, which was induced from the interaction with FliM in a CheY-dependent manner, was counteracted to drive the conformational change of FliG_C_. The E144D/G215A mutation breaks the structure of FliG, preventing the interaction with FliM or FliG by itself, and, consequently, the ability of flagellar formation is lost. The conformational change in FliG_M_ caused by the E144D mutation dominated the conformational change in FliG_C_ caused by the A282T mutation. This suggests that the CW state of FliG_C_ is canceled by the effect of the E144D mutation. We speculated that the Ala to Thr substitution on the A282 residue affects a local conformation around the α helix of FliG_C_^29^. Thus, we can infer that the E144D mutation affects the structure around the α helix.

It is known that serine is the attractant of *Vibrio* and it decreases the switching frequency of the flagellar motor^33,38^. Based on the study of the chemotactic signals in *E. coli*^39^, it was strongly suggested that serine binding to the chemoreceptor promotes CheY-P dephosphorylation through chemotactic signals, so that CheY dissociates from FliM and induces the CCW rotation. To investigate the CheY binding in the C ring in the E144D mutant, we studied the switching events in the E144D mutant after addition of serine. The switching events decreased in the WT under high concentration of serine (2.0 and 5.0 mM); but did not decrease in the E144D mutant. The C ring containing the E144D mutation is supposed to highly fluctuate either in CW state or CCW state even at the native or low concentration of CheY. We found that the E144D mutant in the absence of CheY showed CCW-locked rotation as WT, indicating that it needed CheY binding to FliM to induce the rotational switching. We speculated that association and dissociation constants of CheY from the C ring increased in the E144D mutant and affected CheY binding to FliM and the structure of FliG_M_ and FliG_C_; therefore, E144D mutant can switch frequently with low concentration of CheY.

From the MD simulation and analysis of the conformational ensembles of WT and mutants based on the crystal structure from *T. maritima*, we speculated that the mutations might change the conformation in ARM_M_ and Helix_MC_ on FliG_M_, but the degree of fluctuations might differ among the mutants. The conformations of FliG_C_ in the G214S and G215A mutants may be fixed either in CCW state or CW state by the mutations of FliG_M_, but FliG_C_ in the E144D mutant may fix neither CCW state nor CW state. We assumed that the effect of the interaction between FliG and FliM dominantly determined the rotational direction and switching rather than inducing the conformational change of FliG_M_.

To support the results observed in the phenotype of *fliG* mutant analysis and MD simulation, we measured 2D ^1^H-^15^N TROSY HSQC spectra of the ^15^N-labeled FliG_MC2_ (G122-L351) fragments in WT-FliG and 5 mutations (E144D, Q147H, G214S, G215A, and A218V, which showed tumble, CW-biased, CCW-biased, CW-locked, and CCW-biased rotations). The NMR spectra of WT gave many signals, but less than 229 signals (whole number of fragment), therefore, some signals might be weak, and some could not be detected. This suggests that the WT-FliG_MC_ fragment was largely fluctuating between CW and CCW states in solution states. The spectra of the E144D, Q147H, and A218V mutants were similar to those of the WT, suggesting that these mutants are also fluctuating. In contrast, the significant changes in signal intensities and chemical shift were observed for several amide signals in the G214S and G215A mutations. The signals of the G215A mutation were most sensitive in the WT and other mutations. These results suggested that the G214S and G215A mutations at the Gly-Gly flexible linker tightly fixed the conformations of FliG_MC_, and they supported the results of the MD simulation. In contrast to E144D, the spectra of Q147H and A218V mutant were similar and did not reflect their phenotypes (Fig. S4), suggesting that the conformational changes in FliG_MC_ between the CW state and CCW state were not considerable. We speculated that the Q147H mutation increased the association constant and/or decreased the dissociation constant of between CheY and FliM; thus, the spectrum of the Q147H mutant was similar to that of the WT. We speculated that the structural change caused by the A218V mutation was not so large, but the mutation affected the hydrophobic interaction between ARM_M_ and ARM_C_, and induced the steric hindrance to next molecules in the ring structure of FliG, because the A218V mutation site was located on the surface, important for the hydrophobic interaction.

**Figure 8:**
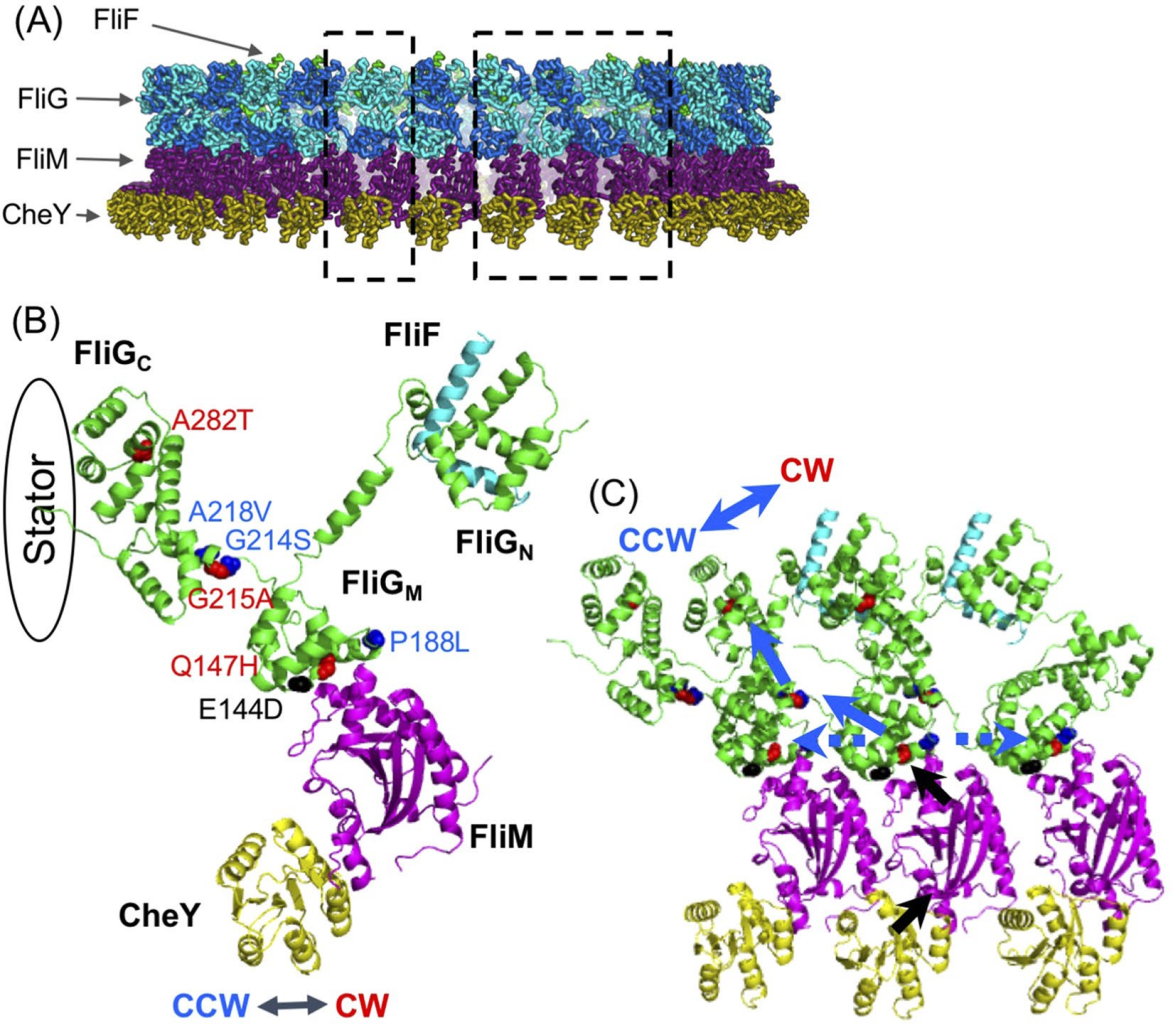
The C ring model of the effects of the mutations in FliG. (**A**) The model of C ring in *Vibrio alginolyticus*. Dashed flames of left and right enlarge 1 set of the C ring (containing one molecule of CheY, FliM, FliG, and FliF) and 3 sets, respectively. (**B**) The mutations in FliG were mapped on 1 set of their components as described in (**A**). Blue, red, and black balls indicate counterclockwise (CCW)-biased rotation, CW-biased rotation, and tumble phenotype, respectively. FliG_C_ interacts with the stator. (**C**) The model of the determination of the rotational direction in the C ring. Black arrows show how CheY binding to FliM affects the EHPQR motif in FliG_M_. Blue arrows show the conformational change in intramolecular FliG. Dashed blue arrows show the effect of the conformational change of FliG on the intermolecular FliG. In our model, CheY binding to FliM induces the conformational change in FliG_M_. This change is sensed by Gly-Gly flexible linker causing the steric hindrance of the conformation of FliG_C_. The conformational change of FliG_C_ affects FliG at both side of ring, being transmitted to the whole FliG of the C ring. Interaction surface between FliG_C_ and the stator slides, so that the rotational switching occurs. FliG in the E144D, Q147H, P188L, and A218V mutants affects FliG at both sides. In contrast, FliG in the G214S and G215A mutants causes the steric hindrance of FliG_C_.

We hereby propose a model for determining rotational direction (Fig. 8) based on the information on *Salmonella* and *E. coli*, CheY binds FliM to change the state from CCW to CW in the C ring (Fig. 8A). The EHPQR motif forms a binding surface for FliM (Fig. 8B). The CheY binding signal must be transmitted to the EHPQR motif to change the conformation. The EHPQR motif seems to have an intramolecular interaction with the Gly-Gly flexible linker region between FliG_n_ and the neighbor molecule FliG_n+1_ in the ring structure of the C ring (Fig. 8C). The conformation of the G214S mutation, which is restricted to the CCW state, is relaxed by the E144D mutation. The E144D mutation caused an increase in the switching events of rotation and exhibited a behavior similar to that observed by the repellent treatment of phenol in WT-FliG. We speculated that the conformational change in FliG_M_ with CheY caused the rapid change of direction and is easy to occur to different directional states in the presence of phenol^40^.

## Material and methods

### Bacterial strains, medium, and growth condition

Bacterial strains and plasmids used in this study are listed in Table S1^23,28,41–50^. To introduce the *cheY* deletion, NMB318 was constructed from NMB198 using pHIDA2, as previous reported^7^. *V. alginolyticus* cells were cultured at 30 °C in VC medium (0.5% [wt/vol] polypeptone, 0.5% [wt/vol] yeast extract, 3% [wt/vol] NaCl, 0.4% [wt/vol] K_2_HPO_4_, and 0.2% [wt/vol] nglucose) or in VPG medium (1% [wt/vol] polypeptone, 3% [wt/vol] NaCl, 0.4% [wt/vol] K_2_HPO_4_, and 0.5% [wt/vol] glycerol). If needed, kanamycin and chloramphenicol were added to a final concentration of 100 μg/mL and 2.5 μg/mL, respectively. *E. coli* cells were cultured at 37 °C in LB medium (1% [wt/vol] bactotrypeptone, 0.5% [wt/vol] yeast extract, 0.5% [wt/vol] NaCl). If needed, kanamycin and chloramphenicol were added to a final concentration of 50 μg/mL and 25 μg/mL, respectively. *E. coli*-introduced pColdI-*fliG*_*MC*_ for overexpression of FliG_MC_ fragment was cultured in M9 medium (0.7% [wt/vol] Na_2_HPO_4_, 0.3% [wt/vol] KH_2_PO_4_, 0.05% [wt/vol] NaCl, 0.002% [wt/vol] bases and vitamins (adenosine, guanosine, cytidine, thymine, thiamine, and biotin), 0.67% [wt/vol] cold-glucose, 0.1% [wt/vol] glycerol, 0.1% [wt/vol] ^15^N-labeled NH_4_Cl, 0.01 mM FeCl_3_, 2 mM MgSO_4_, 0.05 mM MnCl_2_, and 0.1 mM CaCl_2_) containing 100 μg/mL of ampicillin.

### Mutagenesis

To introduce mutations in the *fliG* gene on plasmid pNT1 and pColdI-*fliG*_*MC*_, site-directed mutagenesis was performed using the QuikChange method, as described by the manufacturer (Stratagene). All constructs were confirmed by DNA sequencing. Transformation of *V. alginolyticus* with plasmids pYA303 was performed by electroporation, as described previously^51^. Transformation of *V. alginolyticus* with plasmid pNT1 was performed by conjugational transfer from *E. coli* S17-1, as described previously^41^. Transformation of *E. coli* with plasmid pColdI-*fliG*_*MC*_ was performed by a conventional method.

### Motility assay on soft-agar plate

Cells were grown overnight in VC medium at 30 °C. Approximately 2 μL of cells were spotted on VPG soft-agar containing 0.3% (w/v) agar and incubated for 8 h at 30 °C. We observed the expansion degree of the motility ring.

### Analysis of motile fraction in *fliG* mutants

Cells were grown overnight in VC medium at 30 °C, diluted 1/100 in VPG medium, and incubated for 4 h at 30 °C. Cell suspension (2 μL) was added to 200 μL of V buffer (50 mM Tris-HCl, pH 7.5, 5 mM MgCl_2_, and 300 mM NaCl), and cells were observed by dark-field microscopy. At least 80 cells were observed per condition, and the experiment was repeated three times, for a total of at least 290 cells.

### Investigation of the rotational direction and switching events

The rotational direction and switching events were observed, as described previously^28^. Cells were grown overnight in VC medium at 30 °C, diluted 1/100 in VPG medium, and incubated for 4 h at 30 °C. Cells were washed twice with V buffer. The cell suspension was diluted (1:1) in fresh V buffer or V buffer containing serine. Serine was added to final concentrations of 0.05, 0.1, 0.5, 1.0, 2.0, and 5.0 mM. Motility was observed by high-intensity dark-field microscopy. To avoid the effect of chemotactic adaptation, we observed the samples within 3 min after addition of serine. We measured the rotational direction and switching events in swimming cells for 10 sec. Flagella rotation was determined from the position of the flagellum and the direction of cell swimming. Flagella push the cell body during CCW rotation and pull it during CW rotation. Experiments were performed at least 6 times. For statistical testing, the Welch’s t-test (unequal variances t-test) was conducted for WT-FliG versus the mutants (Figs 2 and 3A,B) or for the V buffer background versus the addition of serine with the V buffer (Fig. 3C). * was indicated the value of *P* > 0.01.

### Observation of polar flagellum in cells

Cells were grown overnight in VC medium at 30 °C, diluted 1/100 in VPG medium, and incubated for 4 h at 30 °C. Cells were washed once with V buffer. The cell suspension was passed through poly-L-lysine-coated tunnel slide with coverslip. To remove extra cells, the glass was washed once with V buffer. To label polar flagellum of the cells, they were incubated in V buffer containing the anti-polar flagellum antibody^52^ for 5 min and were washed twice in V buffer. Cells were incubated in V buffer containing an anti-rabbit IgG conjugated with rhodamine for 5 min. After washing the cells twice with V buffer, the immunolabeled cells were observed using a fluorescence microscope.

### Detection of FliG protein

Cells were grown overnight in VC medium at 30 °C, diluted 1/100 in VPG medium, and incubated for 4 h at 30 °C. Cells were harvested and suspended in distilled water at a cell concentration equivalent to an optical density (OD) of 10 at 660 nm. Proteins were separated by sodium dodecyl sulfate-polyacrylamide gel electrophoresis (SDS-PAGE). Immunoblotting was performed using an anti-FliG antibody (FliGB0164)^53^ as a primary antibody and goat anti-rabbit IgG-HRP as a secondary antibody; the proteins were detected using enhanced chemiluminescence (ECL) Western blotting reagents (GE Healthcare) as the substrate for chemiluminescence and a detector (LAS-3000 mini, Fujifilm).

### MD simulation

The crystal structure of FliG bound to FliM from *T. maritima* (PDB code: 4FHR; Fig. 1A)^20^ for the MD simulations because the structure of FliG of *Vibrio* had not yet been experimentally determined. The region from D113 through M235 (named FliG_MC1_) of *T. maritima* FliG was included in the calculation. Since the region from 188 through 194 was disordered with respect to the structure, the atomic coordinates corresponding to the region were built, and point mutations (E126D, G196S, and G197A) were introduced by using Swiss Pdb Viewer software. Notably, the residues of E144, G214, and G215 in *Vibrio* corresponded to E126, G196, and G197 in *T. maritima*, respectively. After energy minimizations, the obtained structures were used as the initial structures. MD simulations were performed using Gromacs version 4.6.2^54^. The topology was generated with standard amino-acid protonation states at pH 7.0. The force field of AMBER99SB-ILDN and the water model of TIP3P were used for the simulation. The starting structure was placed in a cubic box with 1.2 nm spaces around the solute, and the box was filled with water molecules. Na^+^ ions were added to a concentration of 150 mM. Finally, Cl^−^ ions were added to make the total electrical charge neutral. Time was set at 2 fs. Energy minimization was performed by the steepest descent method. Following energy minimization, the system was equilibrated for 100 ps at 300 K under constant number of particles, volume, and temperature (NVT) and constant number of particles, pressure, and temperature (NPT) conditions. After equilibration, an all-atom MD simulation at 300 K under the NPT condition was performed at 100 ns (5 × 10^7^ steps). Each protein was independently simulated three times. The principal component analysis (PCA) of the MD trajectory was performed by using ProDy^55^.

### The model of C ring structure

The promoter of the full length FliG of *V. alginolyticus* was modeled based on the crystal structures of FliG from *T. maritima* and *A. aeolicus* (PDB code: 3HJL)^12^. The complex structure of FliG (containing FliG_M_ and FliG_C_ domains) and FliM was used as the template structure for the model of FliG_M_ and FliM interaction. The model structure was fitted into the density map of the flagellar basal body from *S. enterica* subsp. (EMDB code: EMD-1887)^56^ to construct the C ring with 34-fold symmetry. The interaction between the FliG_N_ domain and FliN was built according to the complex structure of the FliG_N_ and the C-terminal domain of FliN from *T. maritima* (PDB code: 5TDY)^18^. The interaction between FliM and CheY was inferred on the basis of the complex structure of CheY and the N-terminal fragment of FliM from *E. coli* (PDB code: 1F4V)^57^.

### Purification of *Vibrio* FliG_MC2_ fragment

Cells were grown overnight in 30 mL of M9 medium at 37 °C, inoculated in 1.5 L of M9 medium, and incubated at 37 °C. When the cell density at OD_660nm_ was between 0.38 and 0.45, cells were incubated in ice for 1 h. Isopropyl β-D-1-thiogalactopyranoside (IPTG) was added to a final concentration of 0.5 mM to induce overexpression of FliG_MC_ fragment and inoculated for 1 day at 15 °C. The cells were harvested by centrifugation and stored at −80 °C to break the cell membrane. The frozen cells were suspended with T7.0-N150 buffer (50 mM Tris-HCl, pH 7.0, and 150 mM NaCl) and sonicated using a sonicator (Branson) set on duty cycle 50% and power 5 with proteinase inhibitor, complete EDTA free (Roche Life Science). Unbroken cells were removed by low-speed centrifugation. The samples were ultra-centrifuged at 118,000 × *g* for 30 min. The resultant supernatants were mixed with 5 mg of Talon Metal Affinity Resin (Takara) and incubated at least 10 min at room temperature in a polypropylene column by batch method. After eluting the supernatant in the column, 15 mL (3 fractions of the volume) of T7.0-N150 buffer was added to wash the column. To further wash the column, 5 mL (1 fraction volume) of I30 buffer (50 mM Tris-HCl, pH 7.0, 150 mM NaCl, and 30 mM imidazole) was added. To elute His-tag protein from the resin, 20 mL (4 fraction volume) of I120 buffer (50 mM Tris-HCl, pH 7.0, 150 mM NaCl, and 120 mM imidazole) was added and collected by 1 mL fractions.

The His-tag affinity-purified proteins were concentrated to 1 mL using 10 K Amicon device (Millipore). The samples were subjected to size exclusion chromatography using Superdex 200 Increase 10/300 column (GE Healthcare) in T7.0-N150 buffer with the flow rate at 0.75 mL per min. The peak fractions were collected, and the concentration of samples was measured using Direct Detect spectrometer (Millipore).

### 2D ^1^H-^15^N TROSY HSQC spectra of *Vibrio* FliG_MC2_ fragment

We used the fraction of the highest peak at OD_280nm_ on ^15^N-labeled FliG_MC2_ fragments obtained by size exclusion chromatography whose concentrations were between 0.40 and 0.60 mM. The NMR sample buffer contained 50 mM Tris-HCl, 150 mM NaCl, 0.01% (w/v) sodium 2,2-dimethyl-2-silapentane-5-sulfonate (DSS), and 5% (w/v) D_2_O at pH 7.0. The slotted NMR tube was used, which provided higher signal-to-noise ratio and efficient use of sample mass compared to conventional NMR sample tubes^58^. NMR measurements were performed on an Avance-III HD 500 spectrometer (Bruker Biospin) equipped with a BBO cryogenic probe at 288 K.

In the experiments with 2D ^1^H-^15^N TROSY HSQC^59^ for observing backbone amide NH signals on ^15^N labeled FliG_MC2_ and its mutants, the data size and spectral width were 256 (t1) × 2048 (t2) and 1700 Hz (^15^N) × 7000 Hz (^1^H), respectively. The carrier frequencies of ^15^N and ^1^H were 119 and 4.7 ppm, respectively. The number of scans/FID was 16. All NMR spectra were processed by the Topspin 3.2 (Bruker Biospin).

## Electronic supplementary material

Supplementary Information
