# Supplementary material for: Rotational direction of flagellar motor from the conformation of FliG middle domain in marine Vibrio
Source: Sci Rep. 2018 Dec 12;8:17793. doi: 10.1038/s41598-018-35902-6 (PMC6290876; doi:10.1038/s41598-018-35902-6)
Supplement: Supplementary file 1 — Supplementary Information [file 41598_2018_35902_MOESM1_ESM.pdf]

Supplementary information

Rotational direction of flagellar motor from the conformation of FliG middle domain in marine *Vibrio*

Tatsuro Nishikino, Atsushi Hijikata, Yohei Miyanoiri, Yasuhiro Onoue, Seiji Kojima, Tsuyoshi Shirai, Michio Homma

Table S1. Strains and plasmids used in this study

Strain or plasmid	Genotype or description	Reference or source
V. alginolyticus		
VIO5	Rif^r Pof^+ Laf^-	42
NMB301	VIO5 Δ*pomAB* Δ*fliG* Pof^-	23
NMB198	VIO5 Δ*fliG* Pof^-	51
NMB318	NMB198 Δ*cheY* Pof^-	This study
E. coli		
DH5α	Host for cloning experiments	52
S17-1	*recA hsdR thi pro ara RP-4 2-tc::Mu-Km::Tn7* (Tp^r Sm^r)	53
β3914	*β2163 gyrA462 zei-298::Tn10* (Km^r Em^r Tc^r)	54
BL21(DE3)	F^-, *ompT*, *hsdSB*(r_B– m_B–), *gal*(*λcI* 857, *ind*1, *Sam*7, *nin*5, *lacUV*5-T7*gene1*), *dcm*(DE3)	Novagen
**Plasmids**		
pMMB206	Cm^r, $P_{tac}P_{lac}$UV5	55
pNT1	*fliG* in pMMB206	23
pSU41	Km^r, P_{lac}	56
pYA303	*pomA* and *pomB* in pSU41	57
pSW7848	Suicide plasmid for allele exchange - *oriVR6Kγ oriTRP4 araC*- P_{BAD}-*ccdB*, Cm^r	58
pHIDA2	pSW7848 flanking regions (500 bp) of *cheY*	28
pColdI	Cold-shock expression vector, Amp^r	Takara
pColdI-$FliG_{MC}$	$FliG_{MC}$ fragment (G122-L351) in pColdI	59

Amp^r, ampicillin resistant; Rif^r, rifampin resistant; Cm^r, chloramphenicol resistant; Em^r, erythromycin resistant, Tc^r, tetracycline resistant; Sm^r, streptomycin resistant; Tp^r, thiamphenicol resistant; Pof^+, normal polar flagellar formation; Pof^-, defective in polar flagellar formation; Laf^-, defective in lateral flagellar formation; P_{BAD}, arabinose promoter; P_{trc}, trc promoter

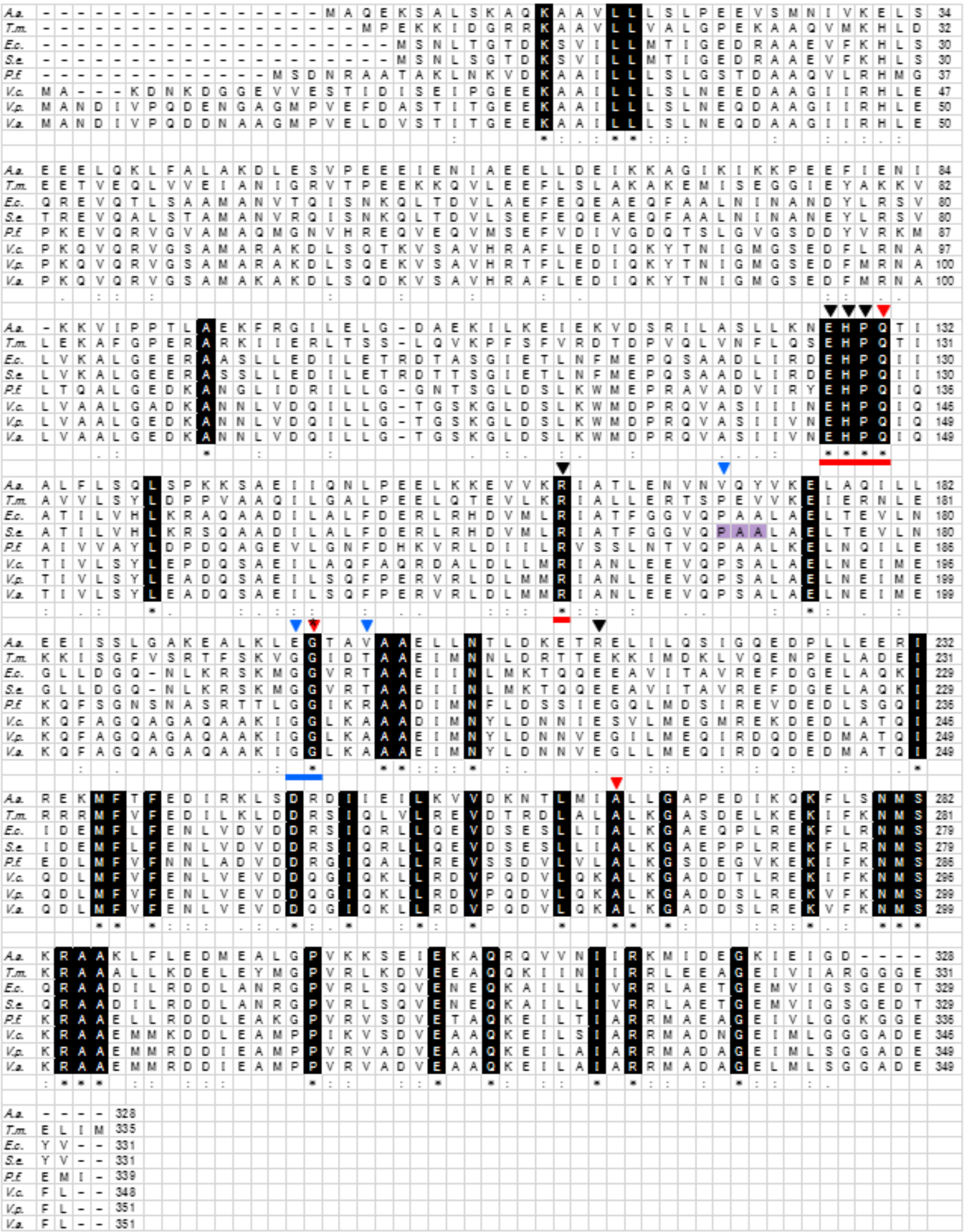

Fig. S1. The alignment of FliG protein of 8 species (*Aquifex aeolicus* (*A. a*), *Thermotoga maritima* (*T.m.*), *Escherichia coli* (*E. c.*), *Salmonella enterica* (*S. e.*), *Pseudomonas fluorescens* (*P. f.*), *Vibrio cholerae* (*V. c.*), *Vibrio parahaemolyticus* (*V. p.*), and *Vibrio alginolyticus* (*V. a.*). The completely conserved residues are showed in the black lane. The EHPQR motif and Gly-Gly flexible linker are underlined with red and blue, respectively. The mutations identified in this study are shown by the arrowhead. Blue, red, and black arrowheads indicate counterclockwise (CCW)-biased rotational mutants, CW-biased rotational mutants, and mutants of other phenotype, respectively. * indicates CW-locked rotation of the G215A mutant in *V. alginolyticus.*

Nishikino *et al.*, Fig. S1

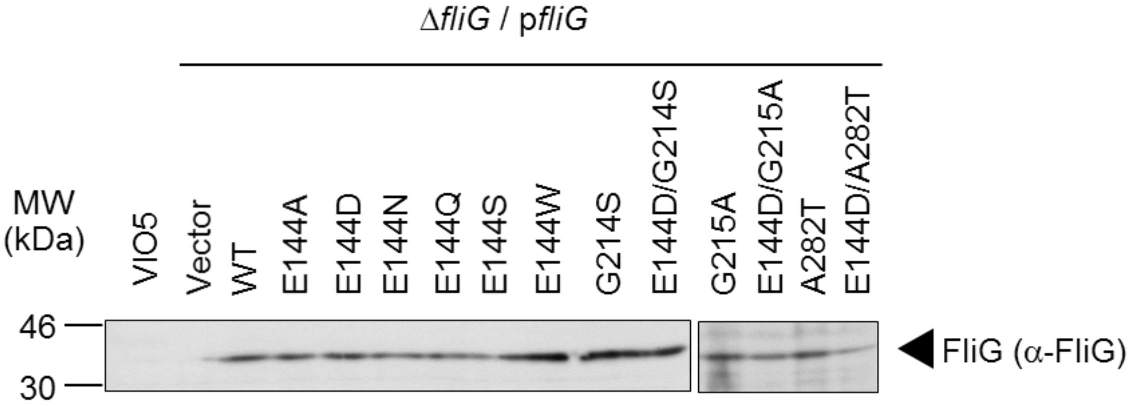

Fig. S2. Detection of FliG protein in *Vibrio* cells. The *fliG/pomA/pomB* mutant (NMB301) harboring pNT1 with a *fliG* mutation and pYA303 with a wild-type (WT)-*pomA* and *pomB* was cultured. Cell concentration was equivalent to an optical density of 10 at 660 nm. FliG protein was detected by immunoblotting using an anti-FliG antibody. The regions of interest were cropped from the immunoblotting.

Nishikino *et al.,* Fig. S2

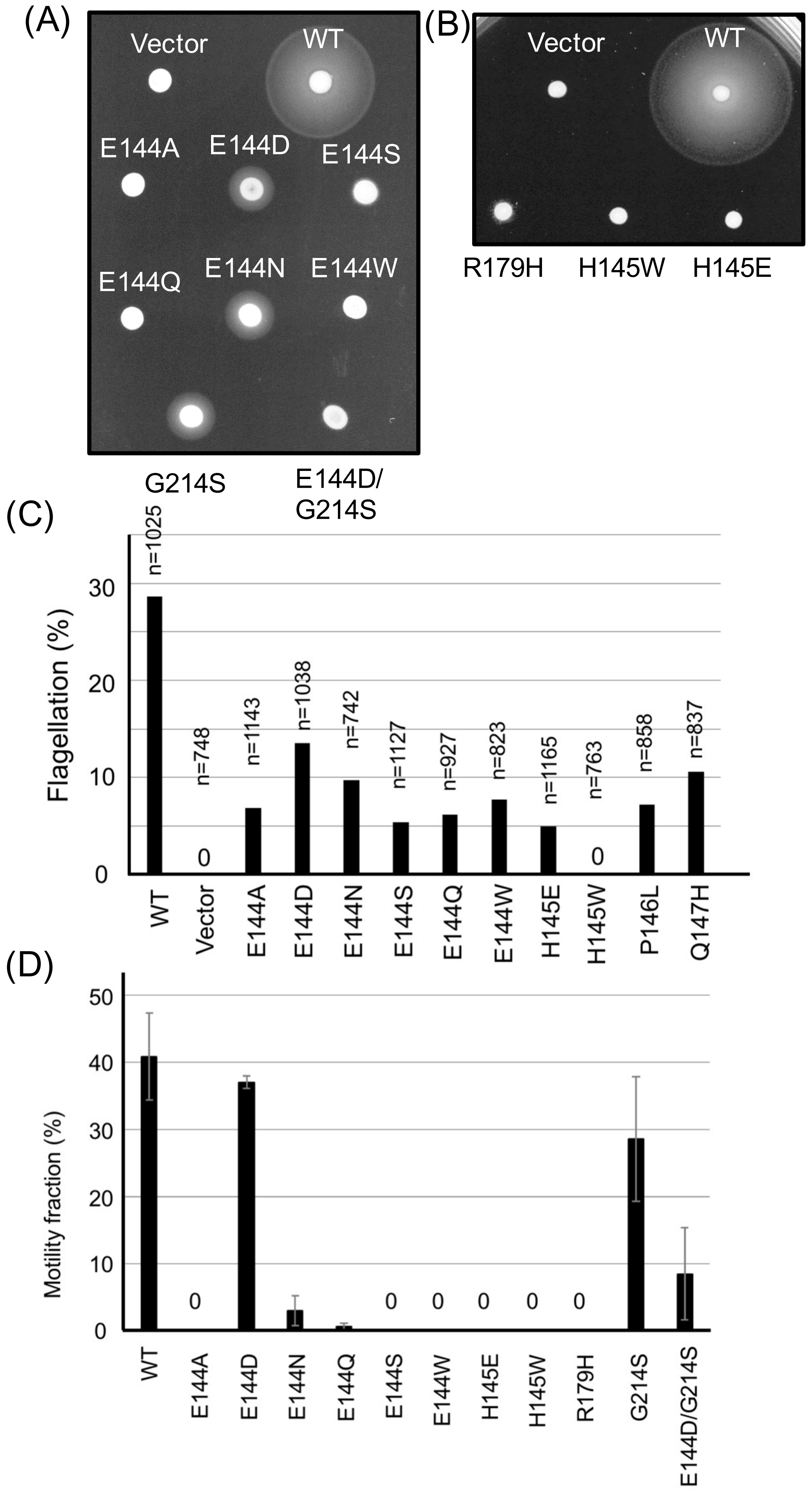

Nishikino *et al.,* Fig. S3

Fig. S3. The EHPQR motif mutants of *Vibrio fliG.* (A, B) The motility assay on soft-agar. The *fliG/pomA/pomB* mutant (NMB301) harboring pNT1 with a *fliG* mutation and pYA303 with a wild-type (WT)-*pomA* and *pomB* was spotted on LB agar (0.3% [wt/vol]) and incubated 8 h at 30 °C. (C) The flagellation fraction of EHPQR motif mutants of *fliG* in *Vibrio alginolyticus*. The *fliG/pomA/pomB* mutant (NMB301) harboring pNT1 with a *fliG* mutation and pYA303 with a WT-*pomA* and *pomB* was cultured. The cells adhered to a poly-L lysine coated glass. The polar flagellum of the cell was labeled with an anti-flagellum antibody and was detected with a rhodamine conjugated secondary antibody. The samples were observed by fluorescent microscopy. The images were obtained using the Health Solutions Plus (HSP) software and analyzed by ImageJ software. (D) The motility fraction of the cells. The *fliG/pomA/pomB* mutant (NMB301) harboring pNT1 with a *fliG* mutation and pYA303 with a WT-*pomA* and *pomB* was cultured and diluted 1/200 with V buffer. The suspensions were observed by dark-field microscopy. Experiments were performed 3 times.

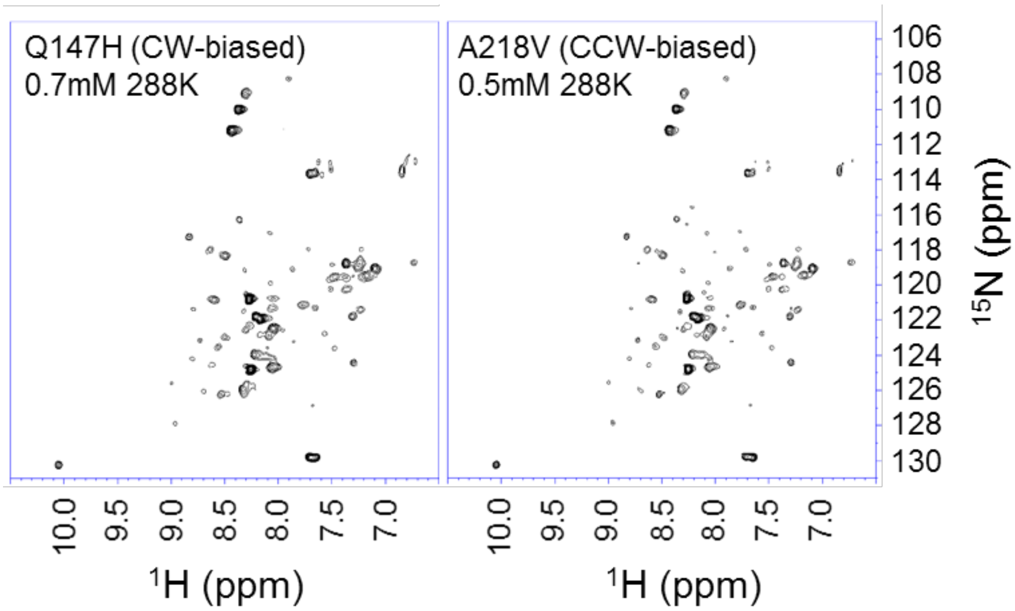

Fig. S4. 2D ^{1}H-^{15}N TROSY HSQC spectra of *Vibrio* $FliG_{MC}$ fragment of the Q147H and A218V mutants. The concentration of ^{15}N labeled sample and the measurement temperature are shown in each spectrum. The mutant phenotype is shown in parenthesis in each spectrum. All spectra were measured by Avance-III HD 500 spectrometer (Bruker Biospin) equipped with a BBO cryogenic probe.

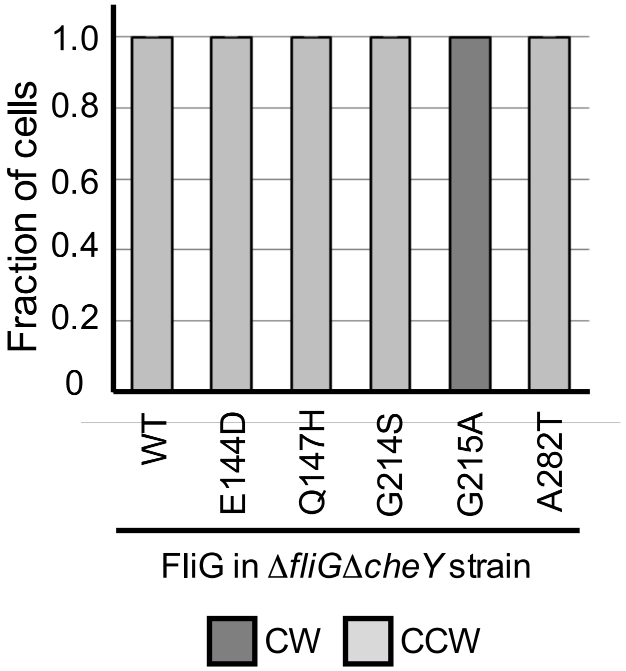

Fig. S5. The rotational direction of *fliG* mutants in the absence of CheY. The *fliG/cheY* mutant (NMB318) harboring pNT1 with a *fliG* mutation was grown and observed by high intensity dark-field microscopy. The ratio of counterclockwise (CCW) rotation to clockwise (CW) rotation of the *fliG* mutants is shown in the column. All the experiments were repeated at least 6 times, and average values with standard deviation (SD) are shown. The switching events per second were zero as all mutants fixed the rotational direction.
